# Male adolescents’ attitude towards justifying wife beating: a study on 20 low and lower-middle-income countries

**DOI:** 10.1186/s12889-025-23088-2

**Published:** 2025-05-23

**Authors:** Nahid Hassan Nishan, M. Z. E. M. Naser Uddin Ahmed, Sayantan Chakraborty, Saidur Rahman Mashreky, Katja Gillander Gådin, Koustuv Dalal

**Affiliations:** 1 Department of Public Health, North South University, Dhaka, Bangladesh; 2 Department of Public Health, Amity Medical School, Amity University Haryana, Gurgaon, Haryana 122412 India; 3 Division of Public Health Science, Institute for Health Sciences, Mid Sweden University, Sundsvall, Sweden

**Keywords:** Attitude, Adolescent attitude, Domestic violence, Justifying wife beating, LMIC, Male adolescent attitudes

## Abstract

**Background:**

This study aimed to examine the prevalence of male adolescents’ attitudes towards wife-beating and to explore the associations between sociodemographic factors and the acceptance of wife-beating in 20 low and lower-middle-income countries.

**Method:**

This study utilized a secondary data analysis from the Demographic and Health Survey (DHS) across 20 lower—and lower-middle-income countries. To understand male adolescents’ attitudes towards justifying wife beatings, we examined factors such as residence, wealth index, education level, and household size. Data analysis was conducted using Stata 17 software, applying weighting methods from the DHS Program to ensure the results accurately represented the target population. Logistic regression was used to examine the relationships between variables.

**Result:**

Among the 26,794 individuals surveyed globally, 4.84% believed that wife beating could be justified. In Guinea, the prevalence stood at 13.42%, whereas Zimbabwe reported a figure of 1.56% in sub-Saharan Africa. Similarly, in Timor-Leste, located in South and Southeast Asia, 16.11% of people justified wife beating, while Myanmar had a mere 2.31% expressing such views. Adolescents residing in rural areas were more likely to endorse wife beating compared to their urban counterparts, who had significantly lower odds (AOR: 0.21, 95% CI: 0.07–0.67). Likewise, higher levels of education played a crucial role in diminishing the likelihood of endorsing wife beating; in Guinea, adolescents with secondary education or higher were substantially less likely to support such attitudes (AOR: 0.32, 95% CI: 0.18–0.58).

**Conclusion:**

The research findings shed light on how young men view violence against women, revealing differences in attitudes across regions. In Sub-Saharan Africa, there are diverse perspectives, with Guinea having a higher prevalence that requires immediate efforts. The concerning prevalence in Jordan highlights the importance of promoting gender equality in West Asia. These findings emphasize the need for interventions that shift beliefs and encourage tangible behavioral changes. Community workshops and skill-building programs can help translate awareness into action. It is crucial to address these attitudes to combat violence effectively.

## Introduction

Wife beating is currently a significant challenge around the globe, and it is also the most common type of violence against women [1]. Understanding the attitudes of male adolescents towards wife beating is crucial, as these attitudes can shape their future behavior as adults. Despite copious research evidence pointing to men as perpetrators of wife beating, most studies examine risk factors that render women vulnerable to their husbands [2]. In low-income and lower-middle-income countries, challenges are often related to economic inequality, gender inequality, and cultural practices perpetuating violence against women. These circumstances can impact the perspectives of men concerning violence.

Since the early 1980s, gender studies have employed the concept of hegemonic masculinity to explain men’s dominance over women. It has been used to explain men’s health behaviors and the use of violence by emphasizing the legitimizing power of consent rather than basic physical or political strength to ensure compliance [3]. A study in Tanzania identified poverty and gender norms as strong predictors of such attitudes. Many poor and lower-middle-income nations have firmly ingrained cultural norms and traditional beliefs, frequently influencing perceptions of gender roles [4]. Studies from India revealed that young men who adhered to conventional notions of masculinity were likelier to justify wife-beating [5].

Another study yielded discoveries that show a relationship between convictions about male power and additional tolerant perspectives among male youths [6]. One more study, which was done in multiple countries in light of South Asian nations, uncovered those young Asian men showed a considerable degree of acknowledgement towards abusive behavior at home. In Bangladesh, 65% of the individuals expressed their support for resorting to violence against women [7]. Similarly, 57% of respondents in India agreed with this notion. However, in Nepal, 28% of the participants agreed with a similar perspective. Various nations have conducted education and awareness campaigns to combat and alter attitudes regarding violence against women [7]. In Ethiopia, it is found that higher levels of education were associated with less acceptance of wife-beating among male adolescents. These findings highlight the potential of education to challenge and transform prevailing attitudes [8]. Also, a study that researched revealed that peer pressure and witnessing family violence were strongly associated with accepting attitudes [9].

Furthermore, research showed that young males who saw their fathers as aggressive were more inclined to excuse wife-beating [10]. Moreover, Educational disparity significantly influences male adolescents’ attitudes, with lower education linked to higher justification of wife beating in low- and lower-middle-income countries [11]. These discoveries stress the requirement for mediations that address these social impacts.

Mentalities towards beating a wife can be affected by the existence of legal systems and methods that promote orientation-based cruelty [12]. In this context, ‘orientation-based cruelty’ refers to legal frameworks or procedures that may perpetuate violence rooted in specific orientations, such as gender, cultural norms, or religious beliefs. Furthermore, a study examines the regulations and surrounding instances of violence against women and those that often assume the involvement of individuals who are not part of the victim’s immediate circle. It is important to recognize that in some cases, despite their behavior, a partner may still maintain a relationship with the woman. Imprisoning the partner could potentially jeopardize the woman and her children’s overall well-being [13]. Thus, understanding male youths’ perspectives towards wife-beating in low and lower-middle-income nations is essential for conceiving successful mediations to battle this cruelty since various countries have their purposes behind the acknowledgement of wife-beating among male adolescents as well as aggressive behavior at home. There may be variations across continents, as adolescents in different regions hold diverse opinions about justifying wife beating. It is essential to address the underlying sociodemographic factors contributing to these attitudes. Therefore, this study aimed to examine the prevalence of male adolescents’ attitudes towards wife beating and explore the associations between sociodemographic characteristics and the acceptance of wife beating in 20 low- and lower-middle-income countries.

## Methodology

### Study design

This study involved a secondary analysis of existing data from the Demographic and Health Surveys (DHSs). The DHS provides comprehensive data from multiple regions, and our focus was on countries classified as Low-income and Lower-middle-income by the World Bank [14]. Initially, we identified 62 countries within this classification. To refine our selection, we applied specific inclusion and exclusion criteria. Countries were included if their datasets were collected between 2012 and 2022, ensuring recency, and if they provided data for all key variables related to our study objectives on attitudes towards the justification of wife-beating. Countries were excluded if the relevant DHS module (e.g., the domestic violence module or men’s questionnaire) was not implemented in the relevant country meaning the key target variables were missing from the country’s dataset. After applying these criteria, 20 countries were found suitable for analysis, providing comprehensive and relevant data aligned with our study’s objectives.

The list of countries that were included in our study are:

Sub-Sahara African Region: Angola 2015-16 [15], Benin 2017-18 [16], Burundi 2016-17 [17], Cameroon 2018 [18], Cote d’Ivoire 2021 [19], Ethiopia 2016 [20], Gambia 2019-20 [21], Ghana 2014 [22], Guinea 2018 [23], Liberia 2019-20 [24], Madagascar 2021 [25], Mali 2018 [26], Niger 2012 [27], Sierra Leone 2019 [28], Togo 2013-14 [29], Zambia 2018 [30], Zimbabwe 2015 [31], South and Southeast Asian Region: Myanmar 2015-16 [32], Timor-Leste 2016 [33], West Asia Region: Jordan 2017-18 [34] Table 1 and 2.

**Table 1:** Demographic table of male adolescents justifying wife beating

Characteristics	Sub-Sahara Africa									
	Angola	Benin	Burundi	Cameroon	Cote d’Ivoire	Ethiopia	Gambia	Ghana	Guinea	Liberia
	*N* (%)	*N* (%)	*N* (%)	*N* (%)	*N* (%)	*N* (%)	*N* (%)	*N* (%)	*N* (%)	*N* (%)
*Residence*										
*Rural area*	351(24.67)	881(59.47)	1431(87.64)	698(46.61)	482(36.15)	2028(80.85)	267(24.39)	441(51.87)	530(56.45)	313(36.58)
*Urban area*	1070(75.33)	600(40.53)	202(12.36)	799(53.39)	852(63.85)	480(19.15)	827(75.61)	409(48.13)	409(43.55)	542(63.42)
*Wealth index*										
*Middle*	242(17.08)	321(21.71)	306(18.74)	345(23.04)	339(25.43)	479(19.11)	189(17.27)	169(19.93)	218(23.25)	186(21.78)
*Poor*	386(27,21)	560(37.81)	536(32.84)	548(36.62)	454(34.08)	803(32.01)	406(37.17)	365(42.85)	276(29.35)	251(29.4)
*Rich*	793(55.71)	600(40.48)	791(48.42)	604(40.34)	541(40.49)	1226(48.88)	499(45.56)	316(37.21)	445(47.4)	418(48.82)
*Adolescent Education*										
*No education*	59(4.15)	310(20.95)	73(4.47)	93(6.19)	191(14.36)	245(9.77)	155(14.19)	21(2.48)	293(31.28)	63(7.23)
*Primary*	484(34.04)	317(21.42)	879(53.81)	341(22.81)	211(15.72)	1737(69.28)	238(21.78)	178(21)	226(24.01)	395(46.27)
*Secondary& above*	878(61.81)	854(57.63)	681(41.71)	1063(71.01)	932(69.92)	526(20.95)	701(64.03)	651(76.52)	420(44.71)	397(46.51)
*Household size*										
*Small (1–3)*	181(12.78)	187(12.59)	195(11.94)	149(9.96)	202(15.15)	309(12.32)	41(3.79)	150(17.6)	32(3.36)	94(11.02)
*Medium (4–6)*	454(31.94)	432(29.14)	623(38.13)	461(30.78)	468(35.13)	1100(43.87)	132(12.08)	417(49.13)	233(24.85)	293(34.25)
*Large (7and more)*	786(55.28)	862(58.27)	815(49.93)	887(59.26)	664(49.72)	1099(43.81)	921(84.14)	283(33.26)	674(71.79)	468(54.73)
*Marital Status*										
*Unmarried*	1420(99.92)	1471(99.32)	1633(100.00)	1495(99.86)	1333(99.92)	2495(99.48)	1094(100.00)	850(100.00)	938(100.00)	855(100.00)
*Married*	1(0.08)	10(0.68)	0(0.00)	2(0.14)	1(0.08)	13(0.52)	0(0.00)	0(0.00)	0(0.00)	0(0.00)

Characteristics	Sub-Sahara Africa							South and Southeast Asia		West Asia
	Madagascar	Mali	Niger	Sierra Leone	Togo	Zambia	Zimbabwe	Myanmar	Timor-Leste	Jordan
	*N* (%)	*N* (%)	*N* (%)	*N* (%)	*N* (%)	*N* (%)	*N* (%)	*N* (%)	*N* (%)	*N* (%)
*Residence*										
*Rural area*	1530(78.91)	603(71.37)	454(69.03)	807(54.14)	513(60.63)	1597(58.34)	1565(73.76)	476(68.68)	548(67.49)	117(11.26)
*Urban area*	409(21.09)	241(28.63)	204(30.97)	683(45.86)	333(39.37)	1141(41.66)	556(26.24)	217(31.32)	265(32.51)	923(88.74)
*Wealth index*										
*Middle*	408(21.04)	172(20.41)	119(18.1)	315(21.17)	173(20.41)	611(22.32)	551(25.97)	160(23.05)	167(20.59)	180(17.29)
*Poor*	619(31.94)	319(37.79)	183(27.8)	486(32.62)	336(39.76)	874(31.92)	738(34.81)	248(35.79)	291(35.76)	356(34.25)
*Rich*	912(47.02)	353(41.81)	356(54.09)	689(46.21)	337(39.83)	1253(45.76)	832(39.22)	285(41.16)	355(43.65)	504(48.47)
*Adolescent Education*										
*No education*	194(9.98)	276(32.67)	243(36.95)	179(12.04)	31(3.56)	72(2.61)	7(0.32)	50(7.22)	63(7.76)	5(0.52)
*Primary*	801(41.36)	187(22.16)	203(30.8)	289(19.4)	157(18.58)	1259(45.99)	561(26.46)	142(20.45)	101(12.43)	54(5.11)
*Secondary& above*	944(48.66)	381(45.17)	212(32.25)	1022(68.56)	658(77.85)	1407(51.4)	1553(73.21)	501(72.33)	649(79.81)	981(94.38)
*Household size*										
*Small (1–3)*	285(14.7)	22(2.65)	44(6.74)	126(8.46)	125(14.74)	227(8.28)	385(18.13)	96(13.79)	69(8.48)	35(3.34)
*Medium (4–6)*	919(47.39)	231(27.35)	153(23.33)	520(34.87)	287(33.95)	893(32.61)	1022(48.2)	401(57.89)	297(36.55)	519(49.91)
*Large (7and more)*	735(37.91)	591(70)	461(69.93)	844(56.67)	434(51.31)	1618(59.1)	714(33.66)	196(28.31)	447(54.97)	486(46.75)
*Marital Status*										
*Unmarried*	1927(99.38)	844(100.00)	658(100.00)	1490(100.00)	846(100.00)	2737(99.96)	2120(99.95)	691(99.71)	812(99.87)	1040(100.00)
*Married*	12(0.61)	0(0.00)	0(0.00)	0(0.00)	0(0.00)	1(0.04)	1(0.05)	2(0.29)	1(0.13)	0(0.00)

**Table 2:** Association and influencing factors between male adolescents towards justifying wife beating in low and lower-middle income

Characteristics	Sub-Sahara Africa																	South & Southeast Asia		West Asia
	Angola	Benin	Burundi	Cameroon	Cote d’Ivoire	Ethiopia	Gambia	Ghana	Guinea	Liberia	Madagascar	Mali	Niger	Sierra Leone	Togo	Zambia	Zimbabwe	Myanmar	Timor-Leste	Jordan
	AOR (95% CI)	AOR (95% CI)	AOR (95% CI)	AOR (95% CI)	AOR (95% CI)	AOR (95% CI)	AOR (95% CI)	AOR (95% CI)	AOR (95% CI)	AOR (95% CI)	AOR (95% CI)	AOR (95% CI)	AOR (95% CI)	AOR (95% CI)	AOR (95% CI)	AOR (95% CI)	AOR (95% CI)	AOR (95% CI)	AOR (95% CI)	AOR (95% CI)
Residence																				
Rural area	Ref	Ref	Ref	Ref	Ref	Ref	Ref	Ref	Ref	Ref	Ref	Ref	Ref	Ref	Ref	Ref	Ref	Ref	Ref	Ref
Urban area	0.31 (0.16–0.59) ***	0.5 (0.17–1.47)	0.72 (0.14–3.64)	1.13 (0.48–2.69)	0.66 (0.21–2.07)	0.36 (0.13–1.02)	0.21 (0.07–0.67) **	1.22 (0.46–3.21)	0.56 (0.27–1.13)	2.38 (0.65–8.79)	2.14 (0.64–7.09)	1.14 (0.37–3.58)	0.6 (0.17–2.11)	0.41 (0.19–0.86) *	0.31 (0.08–1.14)	1.53 (0.75–3.05)	1.37 (0.11–16.24)	0.33 (0.07–1.60)	0.79 (0.43–1.46)	0.48 (0.26–0.86) *
Wealth index																				
Middle	Ref	Ref	Ref	Ref	Ref	Ref	Ref	Ref	Ref	Ref	Ref	Ref	Ref	Ref	Ref	Ref	Ref	Ref	Ref	Ref
Poor	0.76 (0.32–2.02)	1.76 (0.89–3.45)	1.59 (0.50–5.07)	5.32 (1.99–6.17) **	0.96 (0.43–2.13)	0.89 (0.54–1.47)	1.07 (0.35–3.25)	0.65 (0.26–1.65)	0.52 (0.28–0.95) *	0.84 (0.23–3.11)	1.09 (0.49–2.40)	1.55 (0.71–3.38)	0.41 (0.17–0.97) *	0.99 (0.50–1.98)	0.83 (0.28–2.46)	1.34 (0.72–2.49)	1.61 (0.62–4.14)	0.45 (0.11–1.89)	1.06 (0.54–2.10)	0.6 (0.23–1.57)
Rich	1.37 (0.32–5.96)	0.25 (0.07–0.86) *	0.69 (0.18–2.64)	0.82 (0.30–2.25)	0.63 (0.24–1.66)	0.79 (0.47–1.33)	1.28 (0.27–6.27)	0.19 (0.03–1.49)	0.88 (0.46–1.78)	0.77 (0.13–4.44)	0.44 (0.13–1.41)	0.38 (0.15–1.03)	0.59 (0.20–1.70)	1.43 (0.64–3.20)	0.2 (0.05–0.73)	0.49 (0.25–0.96) *	0.09 (0.01–0.77) *	0.94 (0.21–4.28)	0.97 (0.50–1.86)	0.72 (0.32–1.63)
Adolescent education																				
No education	Ref	Ref	Ref	Ref	Ref	Ref	Ref	Ref	Ref	Ref	Ref	Ref	Ref	Ref	Ref	Ref	Ref	Ref	Ref	Ref
Primary	1.01 (0.23–4.36)	0.42 (0.19–0.93) *	0.47 (0.17–1.30)	2.92 (0.77–3.28)	0.54 (0.17–1.67)	0.47 (0.25–0.86) *	2.37 (1.20–4.69) *	1.05 (0.19–5.85)	0.59 (0.32–1.15)	0.29 (0.13–0.69) **	0.33 (0.15–0.69) **	0.76 (0.34–1.71)	1.52 (0.77–2.98)	0.77 (0.36–1.66)	0.81 (0.08–7.47)	3.04 (0.89–10.38)	0.09 (0.01–0.59) *	0.41 (0.07–2.50)	2.11 (0.79–5.89)	0.42 (0.08–2.50)
Secondary & above	0.55 (0.11–2.88)	0.31 (0.15–0.67) **	0.01 (0.02–0.45) **	1.31 (0.34–5.25)	0.73 (0.32–1.69)	0.26 (0.10–0.69) **	0.92 (0.37–2.17)	0.26 (0.07–0.88) *	0.32 (0.18–0.58) ***	0.01 (0.00-0.05) ***	0.17 (0.05–0.49) **	0.84 (0.46–1.64)	1.15 (0.38–3.46)	0.39 (0.20–0.76) **	1.16 (0.15–8.61)	1.51 (0.41–5.46)	0.04 (0.01–0.29) **	0.55 (0.10–3.21)	2.13 (0.90–5.35)	0.89 (0.19–4.10)
Household size																				
Small (1–3)	Ref	Ref	Ref	Ref	Ref	Ref	Ref	Ref	Ref	Ref	Ref	Ref	Ref	Ref	Ref	Ref	Ref	Ref	Ref	Ref
Medium (4–6)	1.22 (0.33–4.53)	0.46 (0.15–1.46)	0.66 (0.21–2.05)	0.37 (0.13–1.11)	3.22 (0.77–13.53)	0.83 (0.40–1.70)	0.45 (0.08–2.72)	1.07 (0.40–2.92)	0.76 (0.24–2.44)	2.61 (0.24–8.37)	4.56 (0.84–5.55)	2.86 (0.55–14.91)	1.01 (0.21–4.62)	1.18 (0.30–4.62)	2.32 (0.29–18.17)	0.81 (0.35–1.85)	1.01 (0.34-3.00)	0.43 (0.08–2.17)	1.51 (0.62–3.68)	0.66 (0.17–2.57)
Large (7 and more)	0.6 (0.16–2.27)	1.14 (0.44–2.92)	1.15 (0.36–3.70)	0.56 (0.23–1.38)	3.72 (0.89–15.47)	0.87 (0.42–1.81)	0.45 (0.09–2.27)	No data	1.08 (0.36–3.30)	13.31 (1.44–17.40)	4.35 (0.84–5.55)	1.81 (0.35–9.34)	1.27 (0.34–4.79)	1.34 (0.31–5.71)	2.12 (0.34–2.96)	0.67 (0.29–0.95) *	0.75 (0.23–2.38)	0.66 (0.13–3.41)	1.19 (0.50–2.84)	0.67 (0.17–2.58)

Denote: (P-value indication: * p-value < 0.05, ** p-value < 0.01, *** p-value < 0.001, AOR = adjusted odds ratio)

### Demographic and health survey

This study analyzed data from the DHS, focusing on the justification of wife beating as recorded in the domestic violence section of the dataset from low- and lower-middle-income countries. The DHS is a globally recognized program that provides nationally representative data on health, population, and social indicators. The DHS aims to support evidence-based decision-making by offering high-quality data on areas like fertility, maternal and child health, gender attitudes, and domestic violence. The DHS employs a two-stage stratified sampling design to ensure representativeness. In the first stage, enumeration areas (EAs) are selected using census data. In the second stage, households within these areas are randomly chosen for interviews. Men aged 15–49 years are then selected from these households as a representative sample. It is important to note that male respondents are not limited to husbands or partners of women surveyed but are selected independently. The Man’s Questionnaire collects data on various topics, including background characteristics (age, education, employment, and marital status), reproductive health (number of children, antenatal care knowledge, and fertility preferences), and family planning (contraceptive use and exposure to related messages). Additionally, it explores men’s attitudes towards gender roles, as well as their knowledge of HIV, stigma, and high-risk behaviors. The DHS ensures data quality through a rigorous process that includes designing country-specific questionnaires, training enumerators, conducting fieldwork, and implementing strict quality control measures. Face-to-face interviews by trained professionals further ensure the reliability of the data [35].

### Study participants

DHS gathers data for households, children, men, and other contexts, but for our study, we focused solely on men’s data, specifically male adolescents aged 15–19 years. This age group was chosen to examine their attitudes towards justifying wife beating. The range was not further categorized, as research shows that this age group represents a distinct and critical stage for understanding the development of long-term beliefs and behaviors. Additionally, DHS provides a separate category for this data, making it a suitable focus for our study [36]. For this study, a total of 26,794 (weighted) participants were suitable among all those continents.

### Outcome variable

The DHS questionnaire assessed whether respondents believed a husband is justified in hitting or beating his wife in specific scenarios, with responses coded as “Yes” for agreement and “No” for disagreement. These scenarios included situations where the wife burns the food, goes out without informing the husband, neglects the children, argues with the husband, or refuses to have sexual intercourse with him. If a respondent agreed with any of these justifications, they were considered to support wife beating, while those who disagreed with all scenarios were categorized as rejecting such justifications. This was used to construct the binary outcome variable for analysis. To construct the outcome variable, respondents who agreed with any of these justifications were coded as 1 (justifying wife-beating), while those who disagreed with all scenarios were coded as 0 (not justifying wife-beating). This binary variable was created using responses from the DHS men’s questionnaire, which includes data collected from men across a wide age range. For this study, we specifically selected responses from males aged 15 to 19 years to focus on adolescent attitudes toward justifying wife beating.

### Covariates

This study examined several sociodemographic variables to explore their association with adolescents’ attitudes towards wife beating. These variables were selected based on insights from the literature and their consistent availability in the DHS dataset across all countries included in the study [2, 37–39]. The age group focused on male adolescents aged 15–19 years, as defined directly in the DHS dataset. Residence was classified as rural or urban based on the DHS question: *“Is your household located in a rural or urban area?”* This variable helps assess the influence of geographic location on attitudes towards wife beating. The wealth index is a proxy measure of household income as a composite measure used in the DHS to estimate household economic status [7, 40]. It is constructed using Principal Component Analysis, which assigns weights to household assets such as ownership of durable goods (e.g., television, refrigerator, bicycle), housing characteristics (e.g., flooring material, roof type), and access to essential services like water supply and sanitation [40]. Based on the resulting scores, households are ranked and divided into five quintiles: poorest, poorer, middle, richer, and richest—each representing 20% of the population [40]. For this study, due to small sample sizes and missing data in some categories, particularly among adolescents, we reclassified the five categories into three: “poor” (combining poorest and poorer), “middle,” and “rich” (combining richer and richest). This grouping ensured sufficient statistical power and is consistent with established DHS methodologies and prior research using similar classification approaches [40, 41]. Education was grouped into three levels: no education, primary education, and secondary or higher education. This was derived from DHS questions on the highest level of schooling completed. Since very few respondents aged 15–19 had completed higher education, the secondary and higher education categories were combined to create a more meaningful analysis. Household size was categorized as small (1–3 members), medium (4–6 members), and large (7 or more members). This variable was based on the DHS question: *“How many people usually live in your household?”* Categorizing household size allowed for an analysis of whether family size influences attitudes towards wife beating. Marital status was determined based on whether the respondent was currently married. Those who had never married or had formerly lived with a woman were grouped as “Unmarried”, while only currently married individuals were classified as “Married”. The DHS collects this information by asking: “Have you ever been married or lived together with someone as if married?” While this study considered key sociodemographic factors, example variables like the sex of the household head were excluded due to inconsistent availability across the DHS datasets for all countries. These factors could provide additional insights but were not feasible for inclusion in this analysis.

### Weighting

In this research, we used the weighting technique to refine the dataset. This approach aims to improve the accuracy of the results and ensure that the survey estimates remain reliable [42]. It is worth noting that different groups within the analysis necessitate different types of weights, such as those for households, women, men, children, and couples. The DHS Program recommended using a method called Weighting Class Adjustment, which corrects for unequal selection probabilities in the sampling design.

For this study, we utilized the individual weights provided for men to ensure that the results accurately represented the population. These weights were scaled as advised by the DHS to account for the sample size and ensure consistency across various countries. Furthermore, the survey design incorporated stratification and clustering, which were addressed using DHS-provided stratification and primary sampling unit (PSU) variables. Stratification divides the population into predefined groups to enhance precision, while clustering considers the multi-stage sampling process employed in household selection. These adjustments were vital to reflect the complex survey design and ensure the analysis was suitable for the male adolescent subsample. Throughout our study, we have meticulously applied weighting techniques to guarantee that our survey sample remains trustworthy and appropriate for analysis.

### Data analysis

To address missing data, we employed a row deletion approach, removing any observations that contained missing values in the essential variables from the dataset. This ensured that the analysis was carried out solely on complete cases, providing a consistent and reliable foundation for our findings. After addressing the missing data, we further refined the dataset by retaining only the necessary variables required for our analysis, concentrating exclusively on those relevant to the study objectives. Regarding the extent of missing data, we found that, on average, less than 1% of the data for the outcome variable was missing across all countries.

We conducted our data analysis using Stata 17 software, ensuring accurate estimates and minimizing bias by accounting for the complex survey design of the DHS dataset. This process incorporated sampling weights, stratification, and clustering of individuals. Binary logistic regression was used to calculate adjusted odds ratios (AORs) with 95% confidence intervals for each covariate related to male adolescents’ justification of wife beating. Statistical significance was denoted using stars (*), with additional stars indicating higher levels of significance: * for p-value < 0.05, ** for p-value < 0.01, and *** for p-value < 0.001. The analysis adjusted for four covariates- residence, wealth index, adolescent education, and household size- in relation to the outcome variable. We also examined the prevalence of adolescents’ justification of wife beating at each country level.

Some countries had a small number of adolescents who justified wife beating (e.g., Myanmar: 16, Ghana: 18, Togo: 18). Even though, we retained these countries in the analysis. The model included a total of eight parameters (seven dummy variables plus the intercept), which constitutes a low-dimensional specification. Each categorical variable was coded using standard dummy variable coding, where a variable with *k* categories contributes *k–1* dummy variables to the model. This approach avoids multicollinearity and enables comparison against a clearly defined reference category, thus ensuring model interpretability and statistical validity [43]. While traditional guidelines for logistic regression recommend a minimum of 10 outcome events per predictor, more recent work supports the use of fewer events per variable (EPV ≥ 2–5) in models that are parsimonious and free of interaction terms or multicollinearity [44]. There were no convergence problems or inflated standard errors in these country-specific models. Additionally, simulation studies have demonstrated that logistic regression models with fewer than 10 cases with the outcome of events per predictor variable can still produce unbiased and stable estimates, particularly when the number of predictors is small and the model is well-specified [44, 45]. Moreover, excluding countries with smaller sample sizes could introduce selection bias and obscure meaningful regional differences in adolescent perspectives on wife beating. Retaining these countries ensures a more accurate representation of geographic variability, particularly in contexts like Myanmar, Ghana, and Togo, where the overall sample size may be limited, but regional insight remains critical to comparative analysis. Additionally, the application of sampling weights in all models helps adjust for unequal probabilities of selection and balances the influence of smaller samples, thereby enhancing the representativeness and comparability of the results across countries [46].

To ensure the reliability of our results, we performed checks for multicollinearity among covariates using a correlation matrix, which showed no evidence of multicollinearity. Adjusted odds ratios (AORs) with their corresponding confidence intervals and significance levels were presented in tables. Model goodness of fit was assessed using the Hosmer-Lemeshow goodness of fit test, with a cut-off point of 10. A p-value greater than 0.05 indicated a good fit for the model [47]. All statistical models for each country demonstrated a good fit based on this criterion.

## Results

### Sample

The demographic characteristics of male adolescents across regions reveal significant variations in residence, wealth index, education, and household size. In Sub-Saharan Africa, Burundi had the highest proportion of adolescents residing in rural areas (87.64%), while Angola had the highest proportion of urban residents (75.33%). Regarding the wealth index, Niger had the largest proportion of adolescents in the rich category (54.09%), while Mali had the highest percentage in the poor category (37.79%). Education levels showed notable differences, with Zimbabwe having the highest proportion of adolescents with secondary education or above (73.21%), while Niger had the highest percentage of adolescents with no education (36.95%). For household size, Niger had the largest proportion of adolescents in large households (7 + members, 69.93%), whereas Angola had the smallest proportion in this category (55.28%).

### Prevalence

**Fig. 1:**
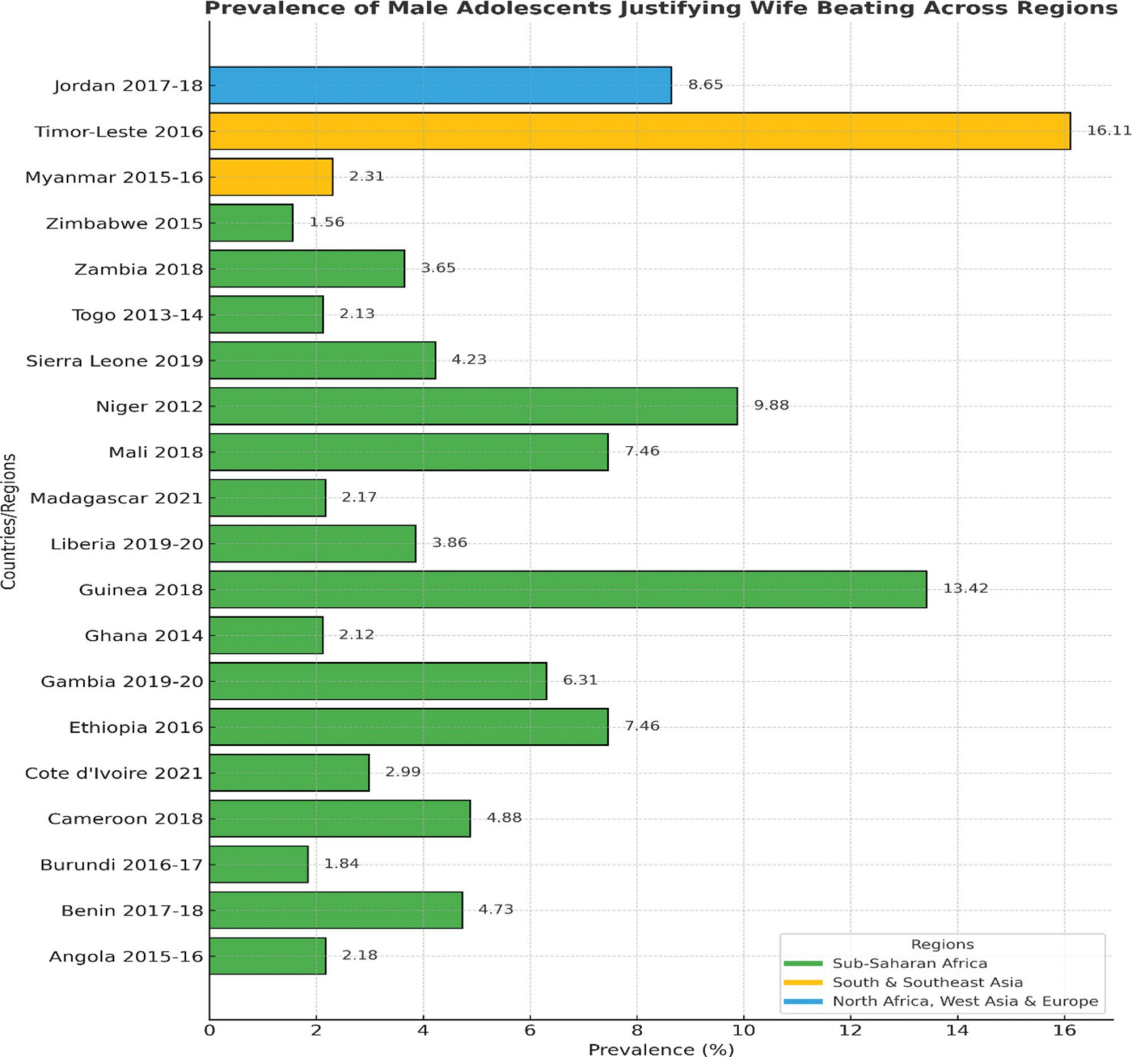
The horizontal bar chart illustrates the prevalence N (%) of male adolescents who justify wife beating in various countries, categorized by region. Colors represent different regions: green for Sub-Saharan Africa, yellow for South & Southeast Asia, and blue for North Africa, West Asia, and Europe. Values are displayed on each bar for clarity

The data in Fig. 1 highlights adolescents’ attitudes towards justifying wife beating across regions, showing both concerning and encouraging trends. In Sub-Saharan Africa, Guinea has the highest prevalence of 13.42%, indicating a need for targeted interventions, while Zimbabwe reports the lowest at 1.56%. Niger’s prevalence of 9.88% underscores the importance of addressing this issue. The regional average for Sub-Saharan Africa is 4.37%.

In South and Southeast Asia, Timor-Leste reports the highest prevalence, at 16.11%, while Myanmar demonstrates a lower prevalence, at 2.31%. The regional average is 9.76%. In West Asia, Jordan shows a concerning prevalence of 8.65% (Fig. 1).

### Associated factors

This study identified key factors influencing male adolescents’ attitudes towards justifying wife beating, revealing distinct trends across various regions. Residence was strongly linked to these attitudes. Adolescents in urban areas were generally less likely to justify wife beating compared to their rural counterparts. In Sub-Saharan Africa, urban adolescents in Angola (AOR: 0.31, 95% CI: 0.16–0.59) and The Gambia (AOR: 0.21, 95% CI: 0.07–0.67) demonstrated significantly reduced odds, as did those in Sierra Leone (AOR: 0.41, 95% CI: 0.19–0.86). Similarly, in West Asia, urban adolescents in Jordan exhibited lower odds of justification (AOR: 0.48, 95% CI: 0.26–0.86).

The wealth index exhibited mixed effects. In Sub-Saharan Africa, adolescents from poorer households in Guinea (AOR: 0.52, 95% CI: 0.28–0.95) and Niger (AOR: 0.41, 95% CI: 0.17–0.97) were less likely to justify wife-beating compared to those in the middle wealth category. Conversely, wealthier adolescents in Zimbabwe showed the lowest odds of justification (AOR: 0.09, 95% CI: 0.01–0.77). However, in Cameroon, poorer adolescents demonstrated higher odds (AOR: 5.32, 95% CI: 1.99–6.17).

Education consistently diminished the likelihood of justification. Adolescents with secondary education or higher in Guinea (AOR: 0.32, 95% CI: 0.18–0.58), Ethiopia (AOR: 0.26, 95% CI: 0.10–0.69), and Zimbabwe (AOR: 0.04, 95% CI: 0.01–0.29) were significantly less likely to justify wife beating. Primary education also displayed a protective effect in countries such as Liberia (AOR: 0.29, 95% CI: 0.13–0.69). Conversely, in Gambia, adolescents with primary education were more likely to justify wife beating (AOR: 2.37, 95% CI: 1.20–4.69), which reflects regional differences.

Household size played a lesser role but remained significant in Zambia. Adolescents from medium-sized households (4–6 members) had lower odds of justification compared to those from smaller households (AOR: 0.81, 95% CI: 0.35–1.85).

## Discussion

This research brings novelty by providing insights into previously understudied regional variations, offering a comprehensive understanding of the multi-continental influences on these attitudes, and focusing on context-specific approaches to combat gender-based violence. Throughout this study, the result provided valuable insight into male adolescent’s attitudes toward wife beating. In Sub-Saharan Africa, the attitudes towards justifying wife beating vary significantly. Guinea stands out with the highest prevalence of people justifying wife beating, which is a cause for concern, and several parameters, including cultural norms, limited access to education and awareness programs, and socioeconomic conditions, could be the main factors for such issues. In contrast, Zimbabwe reports the lowest percentage, indicating a more progressive condition. It is very much crucial for Niger to address issues as their prevalence of 9.88% emphasizes that there could be socioeconomic conditions as the key factor for such a problem. On average, the Sub-Saharan Africa region shows 4.37% justifying wife beating. One possible reason for the variation in attitudes could be the differences in exposure to programs and policies to prevent gender-based violence (GBV) in countries [48].

In contrast, the Guinea population still holds traditions that reinforce dominance over women to the extent of accepting physical violence as a means for a husband to correct his wife, which could explain why adolescents in these countries might have justified wife beating [49]. Another possible cause of such variation is the influence of cultural and religious norms on adolescent’s attitudes toward justifying wife beating. Young people may view wife beating as a form of discipline or correction, while others strongly condemn it as a violation of rights and dignity, which aligns with the previous study within our research findings [50]. In the South and Southeast Asia region. Overall, the region’s average stands at 9.76%, which may highlight the need for awareness campaigns and educational initiatives to challenge harmful beliefs. The higher prevalence of justifying wife beating in Timor-Leste could potentially be attributed to the lasting impact of conflict and violence that has plagued the country.

Conversely, one reason behind the prevalence in Myanmar could be attributed to Buddhism’s influence on how young people perceive violence. Buddhism is a followed religion in Myanmar, where 90% of the people identify themselves as Buddhists. This religion promotes respect for all living beings, compassion towards others, nonviolence, and gender equality. These teachings may have shaped adolescents’ views on GBV, making them less likely to endorse or tolerate justifying wife beating in their relationships [51]. In West Asia, Jordan’s prevalence of 8.65% justifying wife beating is a significant concern in this region, highlighting the necessity of promoting gender equality and combating such attitudes. The global perspective underscores the importance of addressing wife-beating attitudes internationally. Women and girls across the globe encounter obstacles and difficulties that hinder their rights, opportunities, and potential. Additionally, women face higher rates of poverty, violence, exploitation, and oppression in comparison to their male counterparts [52]. These stark inequalities contribute to a pervasive power imbalance between men and women, fostering a culture of domination and subjugation that serves as a crucial backdrop to domestic violence.

The study on adolescent attitudes toward justifying wife beating in Gambia revealed a significant association between residence (rural or urban) and these attitudes. Adolescents in urban areas have a lower likelihood of justifying wife beating towards women, emphasizing the contrast in attitudes between rural and urban people. One potential reason for this correlation could be the variation in media exposure and access to information sources among adolescents residing in urban areas, as this may align with similar study findings [53]. In Guinea, there is a significant association between the wealth index and attitudes toward justifying wife beating. Interestingly, individuals in the middle wealth index category exhibit a reduced likelihood of endorsing attitudes supporting violence against women. This suggests that economic factors can influence attitudes towards justifying wife beating. There are a couple of reasons why this connection might exist. One reason could be that women’s power positively impacts their status and independence within their homes and communities [54]. Another reason could be that when men feel financially insecure, it can affect how they behave and think about women. The research aligns with the relationship between adolescent education and attitudes towards justifying wife beating [55]. It found a significant decrease in the likelihood of supporting violence among educated individuals, emphasizing the role of education in shaping progressive attitudes. One potential reason for this correlation could be the influence of education on the ethical growth of teenagers.

Smaller households may also have more positive role models or mentors from parents or other adults who may condemn or reject justifying wife beating as unacceptable or preventable. These findings highlight that people’s views on violence are multifaceted and influenced by factors such as education, economic situation, and where they live [56]. To tackle these attitudes effectively, it is crucial to implement awareness campaigns and educational initiatives that promote gender equality while discouraging any acceptance or justification of violence. Furthermore, these findings emphasize the significance of comprehending the societal aspects that shape attitudes toward violence in different areas, allowing for tailored interventions to be implemented accordingly.

### Limitation

The primary limitation pertains to the utilization of self-reported data from DHS surveys, potentially compromising data quality and accuracy concerning justifying wife beating and related variables. This could introduce biases and inaccuracies. Furthermore, the perception of attitudes toward wife beating can differ significantly across countries and cultures due to variations in social norms, legal frameworks, and cultural or religious values, which can impact the validity and comparability of data. People from different cultural contexts may interpret survey questions differently, leading to an inconsistent understanding of what constitutes wife beating. Additionally, social desirability bias can influence responses—those in societies where wife beating is condemned might underreport supportive attitudes, while individuals in more permissive contexts may express them more openly. Moreover, cultural differences in the acceptability of certain behaviors can result in discrepancies in the data collected. These emphasize the importance of culturally sensitive survey design and careful interpretation of findings to ensure the data accurately reflects attitudes and remains comparable across different settings. Another limitation is the inconsistency in the time frames of the data sets used for each country, ranging from 2012 to 2022. These variations encompass diverse historical and contextual factors influencing justifying wife beating reporting. Factors like political instability, economic crises, social unrest, natural disasters, or public health emergencies may elevate or diminish violence risks. Additionally, some countries may have implemented legal reforms, policy interventions, awareness campaigns, or support services to address domestic violence, complicating data interpretation. Temporal confounding factors were not considered in the analysis. The unequal sample sizes for each country could affect result precision and representativeness. Countries with larger samples had greater statistical power and reduced sampling error. Moreover, variations in sample diversity among countries could impact result accuracy, undermining the generalizability of findings to each country’s reality. Finally, in the analysis, we had to exclude countries with insufficient data. This limitation affects the generalizability of our findings, like those of Kenya, which also grapples with similar issues. Given these limitations, future studies should consider employing standardized, reliable data sources that consistently capture data across countries and cultures.

## Conclusion

This study provides valuable insights into male adolescents’ attitudes towards gender-based violence, highlighting both encouraging and concerning regional trends. Attitudes vary significantly across regions, emphasizing the need for targeted interventions. In Sub-Saharan Africa, Guinea exhibits a higher prevalence of justifying wife beating, influenced by socioeconomic factors, while Zimbabwe shows more progressive attitudes. Education emerges as a critical factor, with Ethiopian adolescents with higher education levels demonstrating reduced justification of violence. Disparities between rural and urban areas, as observed in Gambia, further underscore the role of sociodemographic factors.

In South and Southeast Asia, Myanmar reflects more progressive views, whereas Timor-Leste highlights the lingering effects of conflict on societal attitudes. Similarly, in West Asia, Jordan’s concerning prevalence of justifying violence against wives underscores the urgent need for initiatives promoting gender equality. Globally, this data underscores the pressing need to address violent attitudes on a critical scale. To effectively combat these attitudes, it is crucial to implement tailored awareness campaigns and educational programs based on the country’s cultural and gender-imbalance context. Understanding and evaluating these factors in different regions is essential for developing targeted interventions that foster more progressive and equitable views towards wife beating.

## Data Availability

Data is available from Demographic and Health Surveys (DHS) program. https://dhsprogram.com.

## Abbreviations

AOR: Adjusted Odds Ratio
CI: Confidence Interval
DHS: Demographic and Health Surveys
EA: Enumeration Area
GBV: Gender-Based Violence

## References

[1] Global and regional estimates of violence against women. https://www.who.int/publications-detail-redirect/9789241564625. Accessed 5 Jan 2024.

[2] Hossain MM, Abdulla F, Rahman A, Khan HTA. Prevalence and determinants of wife-beating in Bangladesh: evidence from a nationwide survey. BMC Psychiatry. 2022;22:9. 34983457 10.1186/s12888-021-03652-x PMC8725961

[3] Jewkes R, Morrell R, Hearn J, Lundqvist E, Blackbeard D, Lindegger G, et al. Hegemonic masculinity: combining theory and practice in gender interventions. Cult Health Sex. 2015;17(Suppl 2):S112–127. 26680535 10.1080/13691058.2015.1085094 PMC4706037

[4] Cislaghi B, Heise L. Gender norms and social norms: differences, similarities and why they matter in prevention science. Sociol Health Illn. 2020;42:407–22. 31833073 10.1111/1467-9566.13008 PMC7028109

[5] Sabarwal S, McCORMICK MC, Subramanian SV, Silverman JG, Son preference and, intimate partner violence, victimization in India: examining the role of actual and desired family composition. J Biosoc Sci. 2012;44:43–56. 21756416 10.1017/S002193201100037X

[6] Barry J. The belief that masculinity has a negative influence on one’s behavior is related to reduced mental well-being. Int J Health Sci (Qassim). 2023;17:29–43. 37416841 PMC10321463

[7] Dalal K, Lee MS, Gifford M. Male adolescents’ attitudes toward wife beating: a multi-country study in South Asia. J Adolesc Health. 2012;50:437–42. 22525105 10.1016/j.jadohealth.2011.09.012

[8] Arefaynie M, Bitew G, Amsalu ET, Kefale B, Muche A, Fentaw Z, et al. Determinants of wife-beating acceptance among reproductive age women in Ethiopia: a multilevel analysis of 2016 Ethiopian demographic and health survey. BMC Women’s Health. 2021;21:342. 34579734 10.1186/s12905-021-01484-1 PMC8474793

[9] Fulu E, Jewkes R, Roselli T, Garcia-Moreno C. Prevalence of and factors associated with male perpetration of intimate partner violence: findings from the UN Multi-country Cross-sectional study on men and violence in Asia and the Pacific. Lancet Global Health. 2013;1:e187–207. 25104345 10.1016/S2214-109X(13)70074-3

[10] Anaba EA, Manu A, Ogum-Alangea D, Modey EJ, Addo-Lartey A, Torpey K. Young People’s attitudes towards wife-beating: analysis of the Ghana demographic and health survey 2014. PLoS ONE. 2021;16:e0245881. 33529235 10.1371/journal.pone.0245881 PMC7853453

[11] Nishan MDNH, Ahmed MZEMNU, Mashreky SR, Dalal K. Influence of spousal educational disparities on intimate partner violence (IPV) against pregnant women: a study of 30 countries. Sci Rep. 2025;15:2022. 39814775 10.1038/s41598-024-84867-2 PMC11736133

[12] Vatnar SKB, Friestad C, Bjørkly S. A comparison of intimate partner homicide with intimate partner homicide-Suicide: evidence from a Norwegian National 22-Year cohort. J Interpers Violence. 2021;36:8231–56. 31104552 10.1177/0886260519849656

[13] Bermúdez Figueroa E, Dabetić V, Yuste RP, Saeidzadeh Z. Gender and structural inequalities from a Socio-Legal perspective. In: Vujadinović D, Fröhlich M, Giegerich T, editors. Gender-Competent legal education. Cham: Springer International Publishing; 2023. pp. 95–142.

[14] World Bank Country and Lending Groups– World Bank Data Help Desk. https://datahelpdesk.worldbank.org/knowledgebase/articles/906519-world-bank-country-and-lending-groups. Accessed 22 Jan 2025.

[15] Instituto Nacional de Estatística. Minstério da Saúde, ICF. Angola Inquérito de Indicadores Múltiplos e de Saúde (IIMS) 2015–2016. Luanda, Angola: INE, MINSA, and ICF; 2017.

[16] Institut National de la Statistique et de l’Analyse. ICF. République du Bénin ciquiéme Enquéte démographique et de Santé Au Bénin (EDSB-V) 2017–2018. Cotonou, Bénin: INSAE/Benin and ICF; 2019.

[17] Ministère à la Présidence chargé de la Bonne Gouvernance et du Plan - MPBGP. Ministère de la Santé Publique et de la Lutte contre le Sida - MSPLS, Institut de Statistiques et d’Études Économiques du Burundi, ICF. Burundi Troisième Enquête Démographique et de Santé 2016–2017. Bujumbura, Burundi: MPBGP, MSPLS, ISTEEBU, and ICF; 2017.

[18] Cameroon DHS. Jan, 2018 - Cameroon 2018 Demographic and Health Survey - Summary Report (English). https://dhsprogram.com/publications/publication-SR266-Summary-Reports-Key-Findings.cfm. Accessed 22 2025.

[19] Institut National de la Statistique, ICF. Côte d’ivoire Enquête démographique et de Santé 2021 rapport final. Maryland, USA et la Côte d’Ivoire: INS et ICF;: Rockville; 2023.

[20] Central Statistical Agency - CSA/, Ethiopia ICF. Ethiopia demographic and health survey 2016. Addis Ababa, Ethiopia: CSA and ICF; 2017.

[21] Gambia Bureau of Statistics - GBoS, ICF. The Gambia demographic and health survey 2019-20. Banjul. The Gambia: GBoS/ICF; 2021.

[22] Ghana Statistical Service - GSS, Ghana Health Service - GHS, ICF International. Ghana demographic and health survey 2014. Maryland, USA: GSS, GHS, and ICF International;: Rockville; 2015.

[23] Institut National de la Statistique, ICF. Guinea demographic and health survey (EDS V) 2016-18. Conakry, Guinea: INS/Guinea and ICF; 2019.

[24] Liberia Institute of Statistics and Geo-Information Services - LISGIS. Minsitry of health - MOH, ICF. Liberia demographic and health survey 2019-20. Monrovia, Liberia: LISGIS/MOH/ICF; 2021.

[25] Institut National de la Statistique (INSTAT). ICF. Enquête démographique et de Santé à Madagascar (EDSMD-V) 2021. Madagascar et Rockville, Maryland, USA: INSTAT, ICF;: Antananarivo; 2022.

[26] Institut National de la Statistique - INSTAT. Cellule de planification et de statistique secteur Santè-Dèveloppement, ICF. Mali demographic and health survey 2018. Bamako, Mali: INSTAT/CPS/SS-DS-PF and ICF; 2019.

[27] Institut National de la Statistique/Niger, Niger UNICEF, International ICF. Enquête démographique et de Santé Dans les zones d’intervention du programme de Cooperation de L’UNICEF Au Niger, 2012. Maryland, USA: INS, Unicef and ICF International;: Rockville; 2013.

[28] Statistics Sierra Leone - StatsSL, ICF. Sierra Leone demographic and health survey 2019. Freetown/Sierra Leone: StatsSL/ICF; 2020.

[29] Ministère de la Planification, Ministère de la Santè - MS/Togo, International ICF. Togo enquête démographique et de Santé 2013–2014. Rockville,Maryland, USA: MPDAT/Togo, MS/Togo and ICF International; 2015.

[30] Zambia Statistics Agency - ZSA, Ministry of Health - MOH. University teaching hospital virology Laboratory - UTH-VL, ICF. Zambia demographic and health survey 2018. Lusaka, Zambia: ZSA, MOH, UTH-VL and ICF; 2020.

[31] Zimbabwe National Statistics Agency, ICF International. Zimbabwe demographic and health survey 2015: final report. Maryland, USA: Zimbabwe National Statistics Agency (ZIMSTAT) and ICF International;: Rockville; 2016.

[32] Ministry of Health and Sports - MoHS/, Myanmar ICF. Myanmar demographic and health survey 2015-16. Nay Pyi Taw. Myanmar: MoHS and ICF; 2017.

[33] General Directorate of Statistics, Ministry of Finance/Timor Leste, ICF. Timor-Leste demographic and health survey 2016. Timor-Leste: GDS and ICF;: Dili; 2018.

[34] Department of Statistics/Jordan, ICF. Jordan population and family health survey 2017-18. Amman: Department of Statistics/Jordan and ICF; 2019.

[35] The DHS Program - Data Collection. https://dhsprogram.com/data/data-collection.cfm. Accessed 18 Jan 2024.

[36] Shinde S, Harling G, Assefa N, Bärnighausen T, Bukenya J, Chukwu A, et al. Counting adolescents in: the development of an adolescent health indicator framework for population-based settings. eClinicalMedicine. 2023;61:102067. 37448809 10.1016/j.eclinm.2023.102067 PMC10336247

[37] Rashid M, Kader M, Perera NKP, Sharma A. Wife beating: A Population-Based study in Bangladesh. Violence Gend. 2014;1:170–5.

[38] ., Department of Population Science, Jatiya Kabi Kazi Nazrul Islam University, Bangladesh, Rashid MM, Rahman M, Department of Population Science and Human Resource Development. Department of Population Science and Human Resource Development, University of Rajshahi, Rajshahi, Bangladesh., Roy TK, University of Rajshahi, Rajshahi, Bangladesh., Sociodemographic Factors Associated With Wife Beating in Bangladesh: Evidence from a Bangladesh Demographic and Health Survey, 2017–2018. IJMRA. 2023;06.

[39] Wang L. Factors influencing attitude toward intimate partner violence. Aggress Violent Beh. 2016;29:72–8.

[40] Rutstein SO, Johnson K. The DHS wealth index. Maryland, USA: ORC Macro;: Calverton; 2004.

[41] Filmer D, Pritchett LH. Estimating wealth effects without expenditure data—or tears: an application to educational enrollments in States of India. World Bank Economic Rev. 2001;15:87–117. 10.1353/dem.2001.0003 11227840

[42] Elkasabi M. Sampling and Weighting with DHS Data. The DHS Program Blog. 2015. http://blog.dhsprogram.com/sampling-weighting-at-dhs/. Accessed 4 Jan 2024.

[43] Gujarati DN, Porter DC. Basic Econometrics. 5th ed. McGraw-Hill Education; 2008.

[44] Vittinghoff E, McCulloch CE. Relaxing the rule of ten events per variable in logistic and Cox regression. Am J Epidemiol. 2007;165:710–8. 17182981 10.1093/aje/kwk052

[45] Van Smeden M, De Groot JAH, Moons KGM, Collins GS, Altman DG, Eijkemans MJC, et al. No rationale for 1 variable per 10 events criterion for binary logistic regression analysis. BMC Med Res Methodol. 2016;16:163. 27881078 10.1186/s12874-016-0267-3 PMC5122171

[46] Heeringa SG, West BT, Berglund PA. Applied Survey Data Analysis. 2nd ed. Chapman and Hall/CRC; 2017.

[47] Bartlett J. The Hosmer-Lemeshow goodness of fit test for logistic regression. The Stats Geek. 2014. https://thestatsgeek.com/2014/02/16/the-hosmer-lemeshow-goodness-of-fit-test-for-logistic-regression/. Accessed 26 Mar 2025.

[48] Muluneh MD, Stulz V, Francis L, Agho K. Gender based violence against women in Sub-Saharan Africa: A systematic review and Meta-Analysis of Cross-Sectional studies. IJERPH. 2020;17:903. 32024080 10.3390/ijerph17030903 PMC7037605

[49] Refugees UNHCfor. Jan. Refworld| Guinea: Domestic violence, including legislation, protection provided to victims and support services (2012 September 2015). Refworld. https://www.refworld.org/docid/563c5fb14.html. Accessed 5 2024.

[50] Islam MM. Child disciplinary practices at home and parental attitudes towards physical punishment to children in Bangladesh. J Child Fam Stud. 2024;33:3904–19.

[51] Chiu Y, Non-Violence. Asceticism, and the problem of Buddhist nationalism. Genealogy. 2020;4:94.

[52] Stamarski CS, Son Hing LS. Gender inequalities in the workplace: the effects of organizational structures, processes, practices, and decision makers’ sexism. Front Psychol. 2015;6. 10.3389/fpsyg.2015.01400 PMC4584998 26441775

[53] Sserwanja Q, Sepenu AS, Mwamba D, Mukunya D. Access to mass media and teenage pregnancy among adolescents in Zambia: a National cross-sectional survey. BMJ Open. 2022;12:e052684. 35701065 10.1136/bmjopen-2021-052684 PMC9198694

[54] Wei W, Sarker T, Żukiewicz-Sobczak W, Roy R, Alam GMM, Rabbany MG, et al. The influence of women’s empowerment on poverty reduction in the rural areas of Bangladesh: focus on health, education and living standard. IJERPH. 2021;18:6909. 34199117 10.3390/ijerph18136909 PMC8293807

[55] Zinyemba KG, Hlongwana K. Men’s conceptualization of gender-based violence directed to women in Alexandra Township, Johannesburg, South Africa. BMC Public Health. 2022;22:2235. 36451124 10.1186/s12889-022-14616-5 PMC9713989

[56] Lanchimba C, Díaz-Sánchez JP, Velasco F. Exploring factors influencing domestic violence: a comprehensive study on intrafamily dynamics. Front Psychiatry. 2023;14:1243558. 37743993 10.3389/fpsyt.2023.1243558 PMC10513418

